# Enhancement of performance and stability of anaerobic co-digestion of waste activated sludge and kitchen waste by using bentonite

**DOI:** 10.1371/journal.pone.0218856

**Published:** 2019-07-10

**Authors:** Ting Zhao, Yongdong Chen, Qing Yu, Dezhi Shi, Hongxiang Chai, Li Li, Hainan Ai, Li Gu, Qiang He

**Affiliations:** Key Laboratory of the Three Gorges Reservoir Region’s Eco-environments, Ministry of Education, Institute of Urban Construction and Environmental Engineering, Chongqing University, Chongqing, PR China; University of Notre Dame, UNITED STATES

## Abstract

There are large amounts of waste activated sludge (WAS) and kitchen waste (KW) produced every year in China. It has been confirmed that anaerobic co-digestion is an effective method to solve this problem. The targets of the present study were optimizing the digestive performances and clearing of the mechanism of bentonite addition by adding bentonite into digestive system. Group M (WAS: KW = 1:2, based on VS) presented higher cumulative methane yield (CMY), where the CMY increased from 19.8 to 36.3 mL/g VS with the bentonite dosage from 0 to 2 g/g VS. After bentonite addition, the lag phase of every digester presented an obvious decrease from 15.1 to 1.4 d. Furthermore, and the moderating effects on microbial community by bentonite. The addition of bentonite improved methane production, and it can also reduce the lag phase of methane production in co-digestion. What's more, bentonite addition increased the speed of pH recovery from 4.2–4.8 to normal level (7.0–8.0) and thus enhanced the system stability. The conclusion of this study can be used to guide practical engineering.

## 1. Introduction

With the rapid development of urbanization, more and more excessive waste activated sludge (WAS) are produced in wastewater treatment process, and it was reported that 55 million tons of sewage sludge (80% moisture) were generated from urban wastewater treatment plants (WWTPs) in China in 2018 [1]. How to sustainably handle these sludge is now becoming a public concern and global environmental challenge [2, 3]. Currently, the costs associated with sludge treatment and disposal account for 40–60% of total operation costs in wastewater treatment [4]. Anaerobic digestion (AD) has been considered as a very well-implemented technology in treating and disposing WAS in WWTP for its considerable environmental and economic benefits [5, 6]. Methane is considered as the major bioenergy that can be drawn from the organic feedstock in AD process. It is also regarded as the most intuitive indicator reflecting organics utilization efficiency. In mono-digestion of WAS, sludge hydrolysis has been identified as a rate-limiting step that depresses the organics utilization and only about 30–50% of organics can be conversed to bio-methane [7, 8]. In addition, WAS in Chinese WWTPs commonly presents low VS/TS ratio ranging 30–60%. So, the insufficient organic contents usually lead to a worse performance in mono-digestion, which eliminates the economic feasibility of bioenergy recovery from sludge anaerobic digestion.

Besides the WAS, kitchen waste (KW) is largely produced in daily life and it is another predominant organic fraction of municipal solid. It is an easily biodegradable biomass with high moisture content [9, 10]. Uncontrolled disposal of KW will cause serious environmental pollution and a waste of resources. KW is composed of high levels of organic substances such as carbohydrate, protein and lipids, which makes it as ideal renewable resource through AD process [3]. However, it is reported that mono-digestion of KW are commonly operated with a relatively low efficiency due to accumulation of VFAs and inhibition of other intermediates [11]. The high biodegradability of carbohydrate fractions in KW leads to a rapid acidification at the initial stage of AD process, which would further inhibit the activity of methanogens, resulting in a long lag phase and a limited methane production.

For the low-organic-contents sludge, the addition of the high-carbon-contents KW would bring a more balance diet and suitable C/N ratios in co-substrate [12], which can improve microbial activity and digestive efficiency. For the KW, due to its high content of easily biodegradable ingredients, it is hydrolyzed rapidly and VFAs accumulation tends to occurs at the initial stage of AD process. When combined with WAS, whose hydrolysis is an alkali-producing process, KW produces more biogas without excessive acidification [13]. Prabhu and Mutnuri (2017) [14] reported that KW mixed with WAS in the ratio of 1:2 produced the maximum biogas of 823 mL/g VS_added_ (21 days) with an average methane content of 60%. Siddiqui et al., (2011) [15] reported that the optimum methane yield of 239 mL/g VS_removed_ occurred at 11% (w/w) of KW in mixture with a C/N ratio of 15, while methane production decreased with the increasing of KW fraction in mixture. It can be drawn from the literatures that the improvement in biogas production and digestive efficiency depend on their mixing ratios, as well as the C/N ratio of the co-substrate [12]. Small proportion of KW in co-substrate mixture can hardly make the maximum effect in improving the biogas production, while too much KW addition would also have risks in VFAs accumulation and hardly maintain the stability of the AD system.

In order to strengthen the process of digestion and the gas production, a series of exogenous materials including biochar [16] and mineral clays [17] are applied in AD process. These additives could be effective as adsorbents or fixing agents in removing or buffering inhibition chemicals such as propionic acid and ammonia. It is reported that the bentonite has a capacity to relieve the inhibitors because of its ion exchange properties and excellent adsorption brought by large surface area [18, 19]. Maqueda et al., (1998) [20] found that bentonite was favorable for start-up of the AD process. Wu et al., (2015) [21] demonstrated that the addition of 10 g/L bentonite achieved faster recovery of inhibited sludge, with a recovery time that was one month shorter than that of the control. However, limited study has investigated the specific buffer ability of bentonite. Based upon analysis, it is expected that the application of bentonite in WAS/KW co-digestion would improve the system stability and promote organics’ conversion to methane, as well as broaden the ranges of mixing ratios of KW and WAS. Higher KW percentages in co-substrate mixture means higher organic fractions in feedstock. It would be of great practical significance in energy recovery that the AD system can keep stable when co-substrate with higher KW fractions are applied. Though there were numerous studies about WAS/KW co-digestion, limited reports can be found in broadening the range of their mass ratio by using bentonite.

Hence, an effort was undertaken in this study to improve the output of WAS/KW co-digestion under mesophilic condition with bentonite addition, aiming to broaden the suitable range of the mix ratio of WAS and KW. In addition, the work was intended to clarify the mechanism of the enhancement effects on WAS/KW co-digestion system in the assistance of bentonite. Specific buffer ability of bentonite were cleared in this study. An in-depth microbial analysis was used to clarify the effects of bentonite addition on microbial community and diversity. The improvement in biogas production, as well as the digestive stability was paid full attention.

## 2. Materials and methods

### 2.1 Substrate and inoculum

KW used in this study was collected from a canteen at Chongqing University, China. It mainly includes rice, vegetables, meat, bones, fishbone, plastic bags, napkin and so on. After picking out bones, fishbone, plastic bags and napkin, it was crushed and stored in a refrigerator at less than 278 K. WAS was taken from Jiguanshi WWTPs of Chongqing, China. It is the biggest WWTPs with an anaerobic-anoxic-oxic (A/A/O) process. Its treatment capacity is about 600 thousand tons per day. Inoculum sludge was the digested sludge discharged from mesophilic anaerobic digester in our laboratory. The digester was fed with WAS with TS 4–6% and had run steadily at a sludge retention time (SRT) of 25 days for more than half year. The digested sludge was sampled and stored under anaerobic condition at 308±1 K for 5~8 days to remove residual organics. The additive bentonite was obtained from Shanghai Test Sihewei Chemical Co., Ltd. The properties of the WAS, KW, inoculum and bentonite are shown in Table 1.

**Table 1:** Characteristics of feedstock components.

properties	Unit	WAS	KW	Inoculum	Bentonite
TS	%	4.03	14.64	2.71	91.99
VS	%	1.48	14.21	1.09	4.62
VS/TS	%	36.72	97.06	40.22	5.02
pH	-	7.2	4.93	7.19	10.61
TOC	% of TS	55.01	64.38	/	0.7
TKN	% of TS	5.64	2.35	/	/
C/N ratios	-	9.75	27.40	/	
Crude protein	%TS	34.21	15.01	/	
Crude fats	%TS	1.6	28	/	
Carbohydrate	%TS	6.21	54	/	

### 2.2 Batch anaerobic digestion experiments

The batch experiments were carried out in a series of 500 mL narrow-mouth glass bottles. These bottles were maintained in a water-bath at controlled temperature (308±1 K). Each reactor was filled with mixture of WAS, KW and IS with different VS ratios. Bentonite was added with specified dosage according the VS amounts of the substrates. The mass ratio of WAS and KW with different bentonite dosages are shown in Table 2.

**Table 2:** Operational parameters of the anaerobic digesters.

	B group					M group					E group				
	B0	B1	B2	B3	B4	M0	M1	M2	M3	M4	E0	E1	E2	E3	E4
VS ratio (WAS: KW)	1:1					1:2					1:3				
Inoculum, mL	100					100					100				
Initial VS, g	8.0					8.0					8.0				
Bentonite dosage, g/gVS	0	0.5	1.0	1.5	2.0	0	0.5	1.0	1.5	2.0	0	0.5	1.0	1.5	2.0
TS, %	6.0					6.0					6.0				
C/N	10.4					11.0					11.7				

Three groups (B, M, E) were set up. The ratios of WAS and KW in B, M and E were 1:1, 1:2 and 1:3 (VS mass ratio), respectively. The amount of inoculum was fixed at 100 mL. There were five digesters in each group, and bentonite dosages for these digesters were set as 0, 0.5, 1, 1.5, and 2.0 g/g VS, respectively. After finishing adding, some distilled water was added to adjust the solid content of the digester to about 6%. The specific experimental scheme is shown in Table 2.

After added, the oxygen of the headspace of the bottles was removed via aerating using nitrogen gas for 10 min. Biogas was collected by using gas sampling bag without any gas washing procedure. The volume of the biogas was measured by using a 250 mL syringe. To monitor the digestion process, digestate was sampled through an outlet at the top of the bottle by using a syringe. Our experiments were carried out in two parallel reactors. Samples were taken from both reactor to analysis different parameters of both slurry and biogas to verify the accuracy of the data.

### 2.3 Analytical methods

Soluble chemical oxygen demand (SCOD), total nitrogen (TN) and ammonia were determined using colorimeter (HACH DR6000, USA) according to the standard methods [22]. The pH was recorded using a pH analyzer (PHS-3S, Leici). TS and VS were determined based on the weighing method after being dried at 378±2 K in the drying oven (QH202A, China) to constant weight and burnt it for 2 h at 873 K in the muffle furnace (CWF1300, China). Biogas composition including methane, hydrogen, and carbon dioxide was determined by a gas chromatograph (Jiedao GC1690, Hangzhou) equipped with a thermal conductivity detector (TCD) and a 5A packed chromatography column (Agilent Technologies, US, 2 m×0.5 mm×0.5 μm). High purity nitrogen was used as the carrier gas at a flow rate of 30 mL/min. The temperatures of the detector and sample injector were 413 K, and the column temperature was 453 K.VFAs compositions were determined by using the same gas chromatograph equipped with a flame ionization detector (FID). DB-FFAP capillary column (30 m×0.32 mm×0.5 μm) was applied in GC. High purity nitrogen was used as the carrier gas. The temperature for the sample injector and the detector is 413 K. The column temperature used temperature programmed mode: initial temperature 373 K, keeping for 3 min, rising at the rate of 6 K/min to 445 K, the final temperature keeping for 2 min.

Crude protein content was calculated by determining total Kjeldahl nitrogen and multiplying by a factor of 6.25 and the total Kjeldahl nitrogen was measured by a Kjeldahl apparatus (K1305A, Shanghai). Crude fat was measured using Soxhlet extraction method. Carbohydrate was calculated by subtracting the amount of crude protein and crude fat from VS [23].

To make a clear understanding the process of methane production, a kinetic assessment of the batch biochemical methane production is applied. The cumulative methane production data were fitted to the modified Gompertz model shown as following:

$$G_{t}=G_{0}\cdot \exp\{‐\exp[\frac{R_{max}\cdot e}{G_{0}}]\cdot (\lambda -t)+1\}$$ (Eq 1)

Where *G*_*t*_ is the volume of cumulative methane (mLCH_4_/g VS) until digestion time *t* (days); *G*_*0*_ is the simulative specific methane yield (mLCH_4_/g VS); *R*_*max*_ is the maximum methane production rate (mL/g VS/day); *λ* is the lag phase (day); *t* is reaction time (day).

Buswell equation was used to analyze theoretical biogas/methane yields and the methane contents in biogas through the elemental fraction of organic substrates [24]. The formula are shown as Eqs 2~5:

$$C_{n}H_{a}O_{b}N_{c}+\left(n-\frac{a}{4}-\frac{b}{2}+\frac{3c}{4}\right)H_{2}O\to \left(\frac{n}{2}+\frac{a}{8}-\frac{b}{4}-\frac{3c}{8}\right)CH_{4}+\left(\frac{n}{2}-\frac{a}{8}+\frac{b}{4}+\frac{3c}{8}\right)CO_{2}+\mathrm{cN}H_{3}$$ (Eq 2)

$$TBY\left[\frac{mL}{g\;VS}\right]=\frac{22415\cdot n}{12n+a+16b+14c}$$ (Eq 3)

$$TMY\left[\frac{mL}{g\;VS}\right]=\frac{22415\cdot (\frac{4n+a-2b-3c}{8})}{12n+a+16b+14c}$$ (Eq 4)

$$Methane\;content[\%]=\frac{TMY[\frac{mL}{g\;VS}]}{TBY[\frac{mL}{g\;VS}]}=\frac{1}{2}+\frac{a-2b-3c}{8n}$$ (Eq 5)

Where TBY represents the theoretical biogas yield (mL/g VS); TMY represents the theoretical methane yield (mL/g VS).

### 2.4 Microbial analysis

After 38 days' digestion, the samples from digester B0, B3, M0, M4, E0 and E4 were selected to analyze the positive effect of bentonite addition in microbial community, respectively. The last day was chosen because related microbes went through a long generation cycle and their dominance were gradually showing out.

Microbial testing services (DNA extraction and PCR amplification, Illumina MiSeq sequencing and processing of sequencing data) were supported by Majorbio Bio-PharmTechnology (Shanghai) Co. Ltd., and specific methods have been shown in our previous study [1].

## 3. Results and discussion

### 3.1 Biogas production

**Fig 1:**
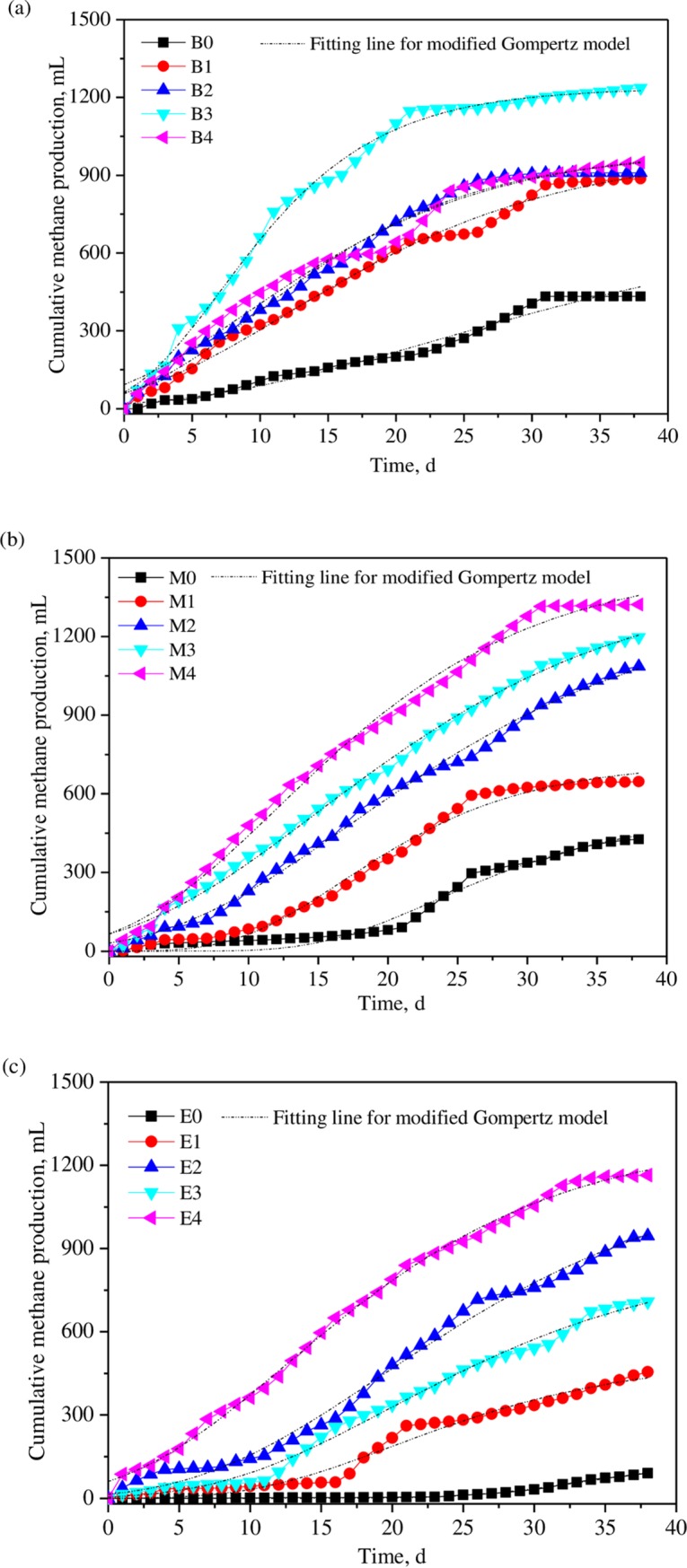
Cumulative methane yield (CMY) and the fitting curve of modified Gompertz model for different groups. (a) B group with the WAS/KW ratio of 1:1; (b) M group with the WAS/KW ratio of 1:2; (c) E group with the WAS/KW ratio of 1:2.

The results of cumulative methane yield (CMY) and the fitting curve for modified Gompertz model for different groups are presented in Fig 1. The parameters obtained in modified Gompertz modelling, as well as the specific methane yield (SMY), specific biogas yield (SBY), average methane contents and VS removal in digestion are presented in Table 3. It can be noticed from the Fig 1 that there was a significant effect of the WAS/KW ratio on methane production. For B0 group whose VS ratio is 1:1 without bentonite addition, the CMY at the final the digestion was about 432.8 mL with a specific methane yield (SMY) of 54.1 mL/g VS. Increasing of the ratio to 1:2 (M0) hardly brought significant changes in CMY, while the methane production curve shown in the figure indicated that the methane production was depressed at the initial stage of the AD process and recovered after 20 days’ digestion. The final CMY for M0 was about 427.2 mL with the SMY of 53.4 mL/g VS. Further increase of the KW fraction in co-substrate mixture (E0, 1:3) brought a total depression of the methane production, and the CMY at the final stage was about 90.4 mL with the SMY of 11.3 mL/g VS. Due to the high fraction of KW in co-substrate, VFA accumulation occurred (as high as 8416 mg/L) and inhibited the activity of the methanogens, resulting in a system deterioration. The result for mono-digestion of WAS can be found from our previous studies [25], while its SMY was about 88.1 mL/g VS. KW mono-digestion would bring a fast system acidification and carbon dioxide dominated in the biogas. Besides methane production, lag phase λ obtained in modified Gompertz modeling can also be applied as an indicator for revealing the process of methane production under different WAS/KW ratios. It can be seen from the Table 3 that the lag phase for B0, M0 and E0 were 5.4, 15.1 and 25.5 days, indicating that the more KW was applied in co-substrate mixture, the longer lag phase in gas production would be.

**Table 3:** Fitting results of modified Gompertz model for accumulative methane yield curves.

Mixture	Digester	Bentonite dosage, g/gVS	Modified Gompertz model				Specific methane yield, SMY	Specific biogas yield, SBY	Average methane contents	VS removal
			*G*_*0*_	*R*_*max*_	λ	*R*^*2*^				
			mL CH_4_	mL CH_4_/d	d		mL CH_4_/gVS	mL/g VS	%	%
Group B WAS:KW = 1: 1	B0	0	721.6±86.7	15.0±2.4	5.4±0.4	0.972	54.1	113.6	47.6	21.5
	B1	0.5	998.4±26.4	31.3±0.4	0.4±0.0	0.993	110.8	207.5	53.4	30.8
	B2	1.0	992.1±16.0	39.4±0.3	0.4±0.0	0.991	113.7	227.8	49.9	28.2
	B3	1.5	1236.6±19.5	69.3±0.2	0.4±0.0	0.992	154.5	258.4	59.8	32.9
	B4	2.0	1013.0±27.9	35.5±0.4	0.3±0.0	0.985	118.6	203	58.4	30.5
Group P WAS:KW = 1: 2	M0	0	521.4±42.2	23.2±0.9	15.1±	0.978	53.4	89.9	59.4	19.8
	M1	0.5	721.3±19.3	34.8±0.3	8.7±0.8	0.982	80.8	151.6	53.3	26.4
	M2	1.0	1324.8±36.3	37.0±0.5	4.3±0.6	0.991	135.7	243.7	55.7	32.0
	M3	1.5	1434.7±33.2	39.7±0.4	1.7±0.1	0.993	149.6	250.9	59.6	33.7
	M4	2.0	1480.7±29.4	50.9±0.3	1.4±0.1	0.994	165.3	270.1	61.2	36.3
Group E WAS:KW = 1: 3	E0	0	189.2±24.2	7.4±1.2	25.5±5.3	0.992	11.3	39.8	28.4	14.8
	E1	0.5	509.7±35.6	19.0±0.9	10.2±1.7	0.979	56.9	144.1	39.5	28.0
	E2	1.0	1203.2±56.7	33.9±0.8	6.3±0.9	0.991	118.2	235.9	50.1	28.7
	E4	2.0	1311.5±17.5	42.9±0.7	1.4±0.2	0.992	145.5	269.8	53.9	38.8
WAS mono-digestion	/	0	691.8±28.3	37.1±1.3	0.2±0.0	0.972	91.3	143.8	63.4	30.1
KW mono-digestion	/	0	107.3±21.5	5.61±0.2	14.6±0.4	0.991	11.7	82.3	14.2	15.7

Performance of methane production under different bentonite addition can also be found in Fig 1. The results shown in this figure revealed the positive effect of bentonite addition in energy recovery and digestive stability. In brief, the application of bentonite in feedstock promoted methane production and maintain the system stability even in digestion of feedstock with high KW fractions. For B group with the WAS/KW ratio of 1:1, with the increase of the dosage from 0.5 to 1.5 g/g VS, the CMY increased from 886.4 to 1236 mL, while the SMY increased from 110.8 to 154.5 mL/g VS, which were 2.0–2.9 times higher than that in WAS/KW co-digestion without bentonite addition. Further increase of the bentonite dosage to 2.0 g/g VS under such ratio depressed the methane production and the SMY was about 118.6 mL/g VS. At the WAS/KW ratio of 1:1, due to the balanced diet in co-substrate, the digestive system was stable and the acids accumulation did not occur. Ma [26] added bentonite to the chicken manure anaerobic digestion system and found that when the system organic load was 19.91g and the bentonite addition is 3% (based on TS), the cumulative methanogenesis was 12% higher than that of the control group. Wang [27] studied the effect of bentonite on the anaerobic digestion process of kitchen waste under different application rates. It was found that the amount of bentonite increased from 1.25% to 2.5% (mass fraction, to digest the substrate wet), the gas production rate of VS decreased by 59.38%. When too much bentonite was added, it would consume a large part of the VFAs through ion exchange, and thus impede the conversion of VFAs into methane due to the reduced VFAs amount in slurry. The lag phase for bentonite addition in B group ranged from 0.3–0.4 days, which were much shorter than that in WAS/KW co-digestion in absence of bentonite. For M group with ratio of 1:2 and E group of 1:3, the SMY increased with the increase of the bentonite dosage without turning point. The highest SMY for M and E group were 270.1 and 269.8 mL/g VS with the bentonite dosage of 2.0 g/g VS. Lag phase for these groups were also calculated and can be found in this table. It can be seen from the figure that the lag phases were all reduced by applying bentonite. For P group, the lag phases for the bentonite dosage of 0.5, 1.0, 1.5, 2.0 g/g VS were 8.7, 4.3, 1.7 and 1.4 days, respectively. The lag phase for E group decreased from 10.2 to 1.4 days with the increase of the dosage from 0.5 to 2.0 g/g VS. The shortened lag phases in digester, especially the digester with high KW fractions, indicated that the bentonite in co-substrate was helpful to maintain the system stability and avoiding fast acids accumulation. Yu [28] found that the addition of powdered activated carbon (PAC) and granular activated carbon (GAC) to the upflow anaerobic sludge blanket (UASB) reactor reduced sludge granulation time by about 30 days and 35 days, respectively. It shows that porous and large surface area materials can accelerate sludge granulation and shorten reactor start-up time. Fagbohungbe [16] observed that biochar addition to citrus peel waste anaerobic digestion not only increased methane production from 163.9 to 186.8 mL CH_4_ g/VS, but also reduced the length of the lag phase to half (7.5 days). Jin [29] found the addition of swine manure digestate hydrochar to the bench-scale pig manure batch digester (TS = 4%) is beneficial to the increase and activity of anaerobic microorganisms, thereby improving the digestion efficiency of the anaerobic fermentation system and shortening the lag phase of anaerobic digestion.

Methane contents in biogas for different digesters during AD process are also presented in this table. WAS mono-digestion presented a relatively higher methane content with the value of 63.4% during its digestive cycle, while the KW mono-digestion presented a much lower methane content with the value of 14.2%. WAS mono-digestion at such initial VS addition can run smoothly, while KW mono-digestion suffered acids accumulation and thus depressed methanogens activities, resulting in limited methane productions and lowered methane content in biogas. It is also noticed from this table that the bentonite addition not only promoted methane production, but also improved the methane contents of the biogas. Buswell formula was commonly used to explain the variation of methane contents in digestion of different substrates. According to the formula, higher fractions of carbon (C) and oxygen (O) elements in VS result a decline in methane content. Hence, the WAS mono-digestion presented the highest methane contents due to abundant carbohydrate WAS. Though KW is also a substrate rich in nitrogen, its high biodegradability fractions usually leads to a rapid acidification, which hinders the methanogens activities and thus lowered the methane production, as well as methane contents. Nevertheless, under the same VS addition amount, the methane contents in biogas varied with bentonite dosages. So, it can be drawn that the bentonite assists organics’ decomposition, as well as their conversion to methane. The VS removal for different digesters are shown in Table 3.

VS removal efficiency is an important index evaluating AD performance. High VS removal means that more organic substances are utilized by microorganism and more biogas may be produced. As shown in Table 3, WAS/KW co-digestion presented lower VS removal varying from 14.8 to 38.8% depending on their ratios. Though there was a much longer lag phase for M0 than that in B0 in methane production, the digester M0 presented the highest VS removal at the end of the digestion, which can be ascribed to the more balanced nutrient in co-substrate under the ratio of 1:2. E0 presented the lowest VS removal due to VFAs accumulation, which hindered their further degradation or conversion. Bentonite-added digester presented much higher VS removal compared to non-bentonite-added ones. For B group, the highest VS removal (B3, 32.9%), which was 1.98 times higher than that in B0, appeared with the bentonite dosage of 1.5 g/g VS, and further increase of the dosage to 2.0 g/g VS brought a slight decline of the VS removal to 30.5%. For M and E group, the highest VS removal appeared under the dosage of 2.0 g/g VS, while their values were 33.7% and 38.8%, respectively. Dang [30] found that the addition of conductive materials (carbon cloth or granular activated carbon (GAC)) to the anaerobic digester treating dog food increased the removal rate of volatile solids (VS) from 54%-64% to 78%-81%.

### 3.2 Digestate characterization

#### 3.2.1 pH value

**Fig 2:**
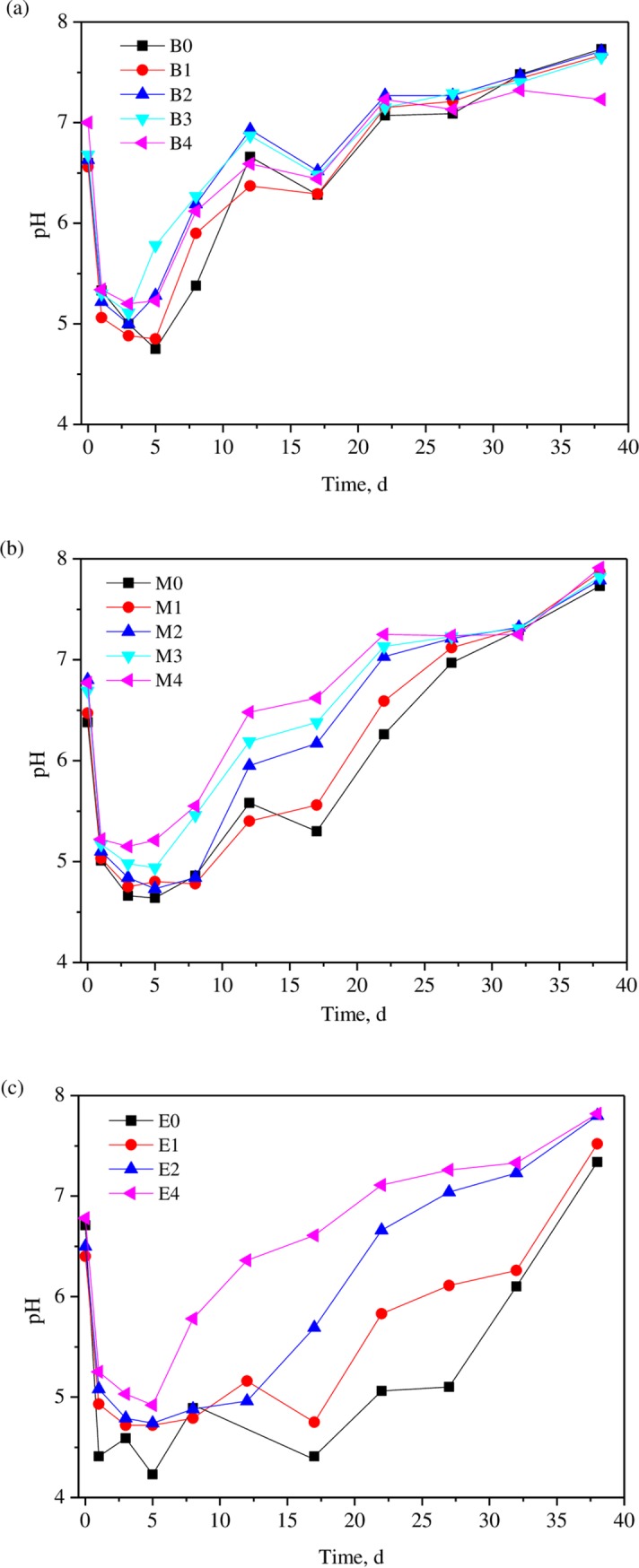
Variations of pH in anaerobic digesters.

It is reported pH is one of the key parameters representing stability of AD system, and the pH variation for different trials are shown in Fig 2. It can be seen from the figure that during the first 3–5 days, the pH for all trials declined due to the fast-hydrolytic acidification process caused by decomposition of perishable organics substances. At this time, few of biogas was produced. It can be seen from this figure that, for the non-bentonite-added digesters, higher KW fractions in co-substrate mixture brought a steeper decline of the pH. For the substrate with the WAS/KW ratio of 1:1 (B group), the lowest pH appeared after 5 days’ digestion with value of 4.8, while that for M and E groups were 4.6 and 4.2. In AD process, pH value commonly recovers after the rapid drop due to the conversion of the acids into biogas, while the recovery process were obvious in both B0 and M0 digesters. However, the pH in E0 digester did not recover soon afterwards and the value remained lower than 5.0 until 23^rd^ days. It is widely acknowledged that the digesters’ pH can affect microorganisms’ activity, while different microorganisms favor different optimal pH ranges [31]. Methanogens work effectively at pH varying from 6.5 to 8.2, and acidogens work effectively at pH varying from 5.2 to 6.5. Hence, the maintenance of pH ranging 4.2–5.0 during the first 15 days’ digestion depressed methanogens’ activities and inhibited the conversion of acids to biogas, resulting in a long lag phase of methane production and an acids accumulation during early and mid-stage of AD process.

When bentonite was added into the digester, the situation was changed. There are negative charges on bentonite surface due to exchangeable cations in its structural layers. These cations, including Na^+^ and Ca^2+^, involved in formation of outer-sphere surface complexes, can be exchanged by H^+^ ions and thus presents a strong buffering ability towards organic acids [32]. It can be seen from the Fig 2 that though the pH in bentonite-added digesters varied as the same paths with non-bentonite-added digesters, higher pH values were obtained in this group for all digesters, showing the buffering effect of bentonite on acids accumulation. The addition of bentonite not only increase the bottom pH value of the digesters, but also enhanced self-recovery ability of the digesters. The more bentonite was applied, the higher recovery rate can be observed. Take E group (WAS/KW = 1:3) for example, when 2.0 g/g VS bentonite was applied (E4), pH of digester was about 4.9, which was much higher than that in E0 digester (pH = 4.2). Furthermore, during the latter 18 days, the pH for E0 digester remained lower than 5.0, while that in E4 digester (bentonite dosage 2.0 g/g VS) increased from 4.9 to 7.1. So, the applications of bentonite brought suitable pH conditions for methanogens, and the conversion of organics to methane can be accelerated and proceeded much more smoothly, resulting in a shortened lag phase and improved conversion efficiency. Ma [26] and Pan [33] also found that the addition of bentonite to the anaerobic digestion of kitchen waste has a certain buffering effect on the change of pH and this buffering effect relatively obvious in the early stage of the digestion process (1~7d).

#### 3.2.2 VFAs’ concentrations

**Fig 3:**
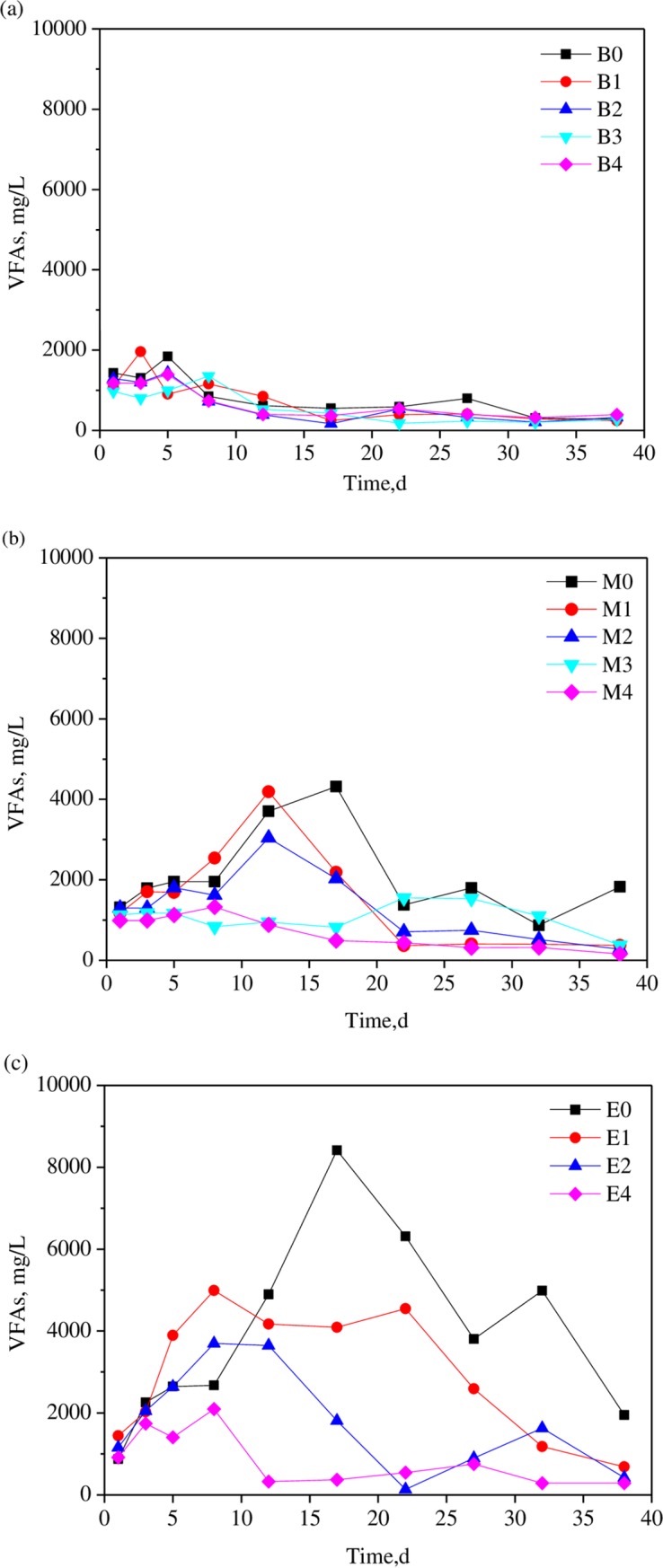
Variation of VFAs’ concentrations for different trials.

Fig 3 shows the VFA concentration profile during the AD process in different trials. Comparison among B, M and E groups showed that the VFAs’ concentrations were elevated by the increased KW fractions in co-substrate mixture. For B group with the WAS: KW ratio of 1:1, the VFAs’ concentrations for all the digesters in B group were below 2000 mg/L. For M group with the ratio of 1:2, the highest VFAs’ concentration with the value of 4318 mg/L appeared after 18 days’ digestion in M0 digester without bentonite addition. It is clear that the addition of bentonite not only decreased the VFAs’ concentrations, but also brought forward of the appearance of the peak VFAs’ concentration (digester M1 and M2 with the bentonite dosage of 0.5 and 1.0 g/g VS). It should be noticed that at the higher bentonite dosage of 1.5 and 2.0 g/g VS, the VFAs’ concentrations retained at lower than 2000 mg/L throughout the digestion process. In addition, when the WAS: KW ratio achieved to 1:3 (group E) in which KW dominated in the co-substrate, much higher VFAs concentrations can be obtained. The peak TVFAs’ concentration for WAS/KW co-digestion (without bentonite addition) appeared after 16 days’ digestion, and its value was 8416 mg/L, which was much higher than other groups. The high TVFA concentrations in digester indicated that VFAs accumulated and system acidification occurred inevitably. So, the SMY in E0 digester was only about 11.3 mL CH_4_/g VS with a limited methane content in biogas. Bentonite addition in E group presented stronger buffering ability to that in B and M groups. Under the bentonite dosage of 0.5, 1.0 and 2.0 g/g VS, the peak TVFAs concentrations were decreased to about 4990, 3698 and 2094 mg/L. In addition, the TVFAs concentration peaks appeared after 8 days’ digestion, which were 9 days earlier than that in E0 digester. These results show that the application of bentonite in WAS/KW co-digestion could neutralize excessive VFAs in digester, and thus stabilize anaerobic system. Zhang [34] also found that adding bentonite to the anaerobic digestion of kitchen waste can prevent volatile fatty acids (VFA) accumulation and obtain a high methane production at low OLR (1.39 gVSL^-1^ d^-1^).

### 3.3 Mechanism study

#### 3.3.1 Bentonite’s adsorption ability

**Fig 4:**
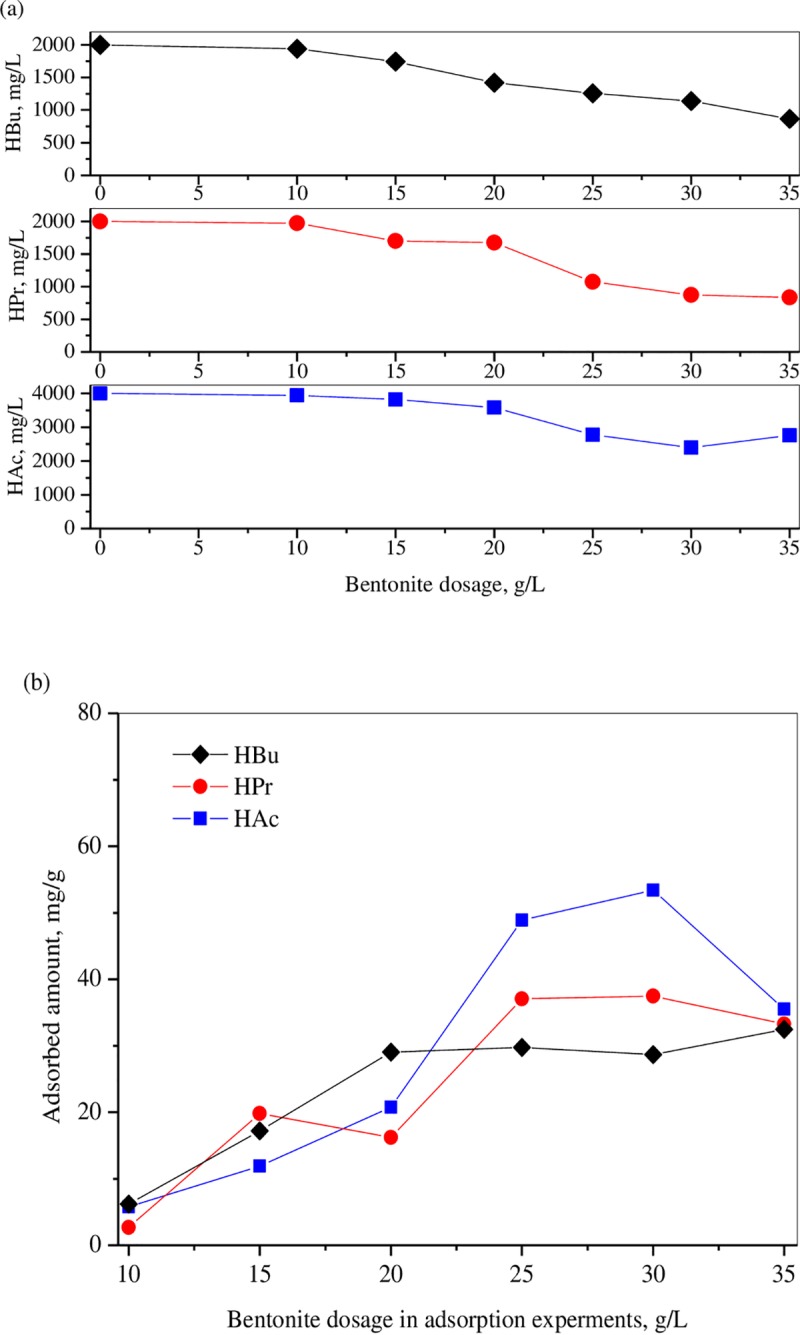
Adsorption properties of bentonite towards different acids. (a) Variation of the final VFAs’ concentrations under different bentonite dosage; (b). Variation of adsorbed VFAs’ amounts under different dosages).

To reveal mechanism of the promotion in methane production and systematic stability in AD system, adsorption properties of bentonite for three types of VFAs including acetic acid (HAc), propionic acid (HPr) and butyric acid (HBu) were examined. According to the results obtained in the analysis about the VFAs’ concentrations, the initial HAc concentration in adsorption experiments was set as 4000 mg/L, while that for HPr and HBu were set as 2000 mg/L. The results can be found in Fig 4. From the figure, it can be seen that the bentonite present good adsorption ability to the selected acids. For HAc solution with an initial concentration of 4000 mg/L, the bentonite presented a limited adsorption ability at lower dosages. The HAc concentration at the end of the adsorption process under the dosage of 10, 15 and 20 g/L were 3942, 3821 and 3585 mg/L. Further increase of the dosage to 30 g/L brought significant decrease of the final concentration to about 2398 mg/L. The adsorbed amounts for different bentonite dosage can also be found in the Fig 4B. The maximum adsorption amount of bentonite for HAc was about 53.4 mg/g. For HPr, the final concentration was decreased gradually to about 86.0 mg/L with the increase of the bentonite dosage, while the maximum adsorption amount was about 37.5 mg/g. It is reported that HPr was a dominant VFA in inhibiting the activity of methanogens, and the tolerance concentration of HPr to methanogens is about 1000 mg/L [35]. So, the addition of bentonite can effectively control HPr concentrations, and reduce its potential opportunity in inhibition of methanogens, resulting in the improvements in methane production performance. Compared to HPr and HAc adsorption, bentonite presented a weaker affinity to HBu, and the lowest concentration at the end of the adsorption was about 863.7 mg/L, corresponding to the maximum adsorption amount of 32.5 mg/g. Based upon analysis, the coalescent effect between bentonite and VFAs is confirmed to have positive effect on AD process. The bentonite decrease the VFAs concentrations through adsorption effect, which avoids fast accumulation of VFAs and maintains suitable environments for methanogens. In addition, it should be noticed from Fig 3 that in the E group with much higher TVFAs concentrations, the reduction amounts of TVFAs brought by bentonite addition varied from 3426 mg/L (E1) to 6322 mg/L (E4), depending on the bentonite dosage. However, these VFAs reduction amounts were far more than the adsorption capacities of bentonite, which can be calculated from the maximum adsorption amounts shown above. Therefore, it can be concluded that, besides the physical/chemical effect brought by bentonite adsorption, the bentonite also posed positive effects in maintaining the stability of biological communities, by which both the stability and the efficiency of AD process were improved.

#### 3.3.2 Analysis of the microbial community compositions

The different substrates ratios can change the digestive conditions and thereby affect the microbial diversity, and the methane production performances will be changed. After subsampling, each sample has an equal sequencing depth (30852 reads per sample) shown in Table 4. Among all index, Sobs, Chao and Ace are used to represent community richness, while Simpson and Shannon are used to represent microbial diversity. From the community richness analysis in Table 4, it can be seen that bentonite-added groups presented higher richness than non-bentonite-added groups of every trials. Generally speaking, a higher community richness reflects a better ecological stability and a higher ability in resisting toxic in AD process [36], which could be used to illustrate the reason that bentonite-added groups presented a more stable system than non-bentonite-added groups. However, in terms of microbial diversity, it can be seen from Simpson and Shannon index that bentonite-added groups presented less microbial diversities, indicating the addition of bentonite led to selection of microbes in digestive system. Sasaki [37] observed that addition of the carbon fiber textiles (CFT), stabilized the thermophilic methanogenic bioreactors even at higher total ammonia nitrogen (TAN) concentration up to 3000 mg/L. The retention of the higher number of methanogenic archaea at the CFT is Methanosarcina. In our research, all the Coverage is near to 1.0, showing that the sequencing results can represent the real situation of the microorganisms in the sample. Detailed microbial community analysis is shown below.

**Table 4:** Biodiversity estimation of 16S rRNA gene libraries of bacterial community.

Parameters	Sobs	Chao	Ace	Simpson	Shannon	Coverage
B0	557	657.44	655.03	0.039	4.30	0.997
B3	582	671.77	627.61	0.029	4.37	0.997
M0	557	647.28	668.32	0.102	3.60	0.996
M4	567	696.00	632.41	0.075	3.78	0.996
E0	562	626.60	664.26	0.072	4.09	0.997
E4	579	690.05	619.15	0.060	4.00	0.996

(1) Pyrosequencing analysis of bacterial community

**Fig 5:**
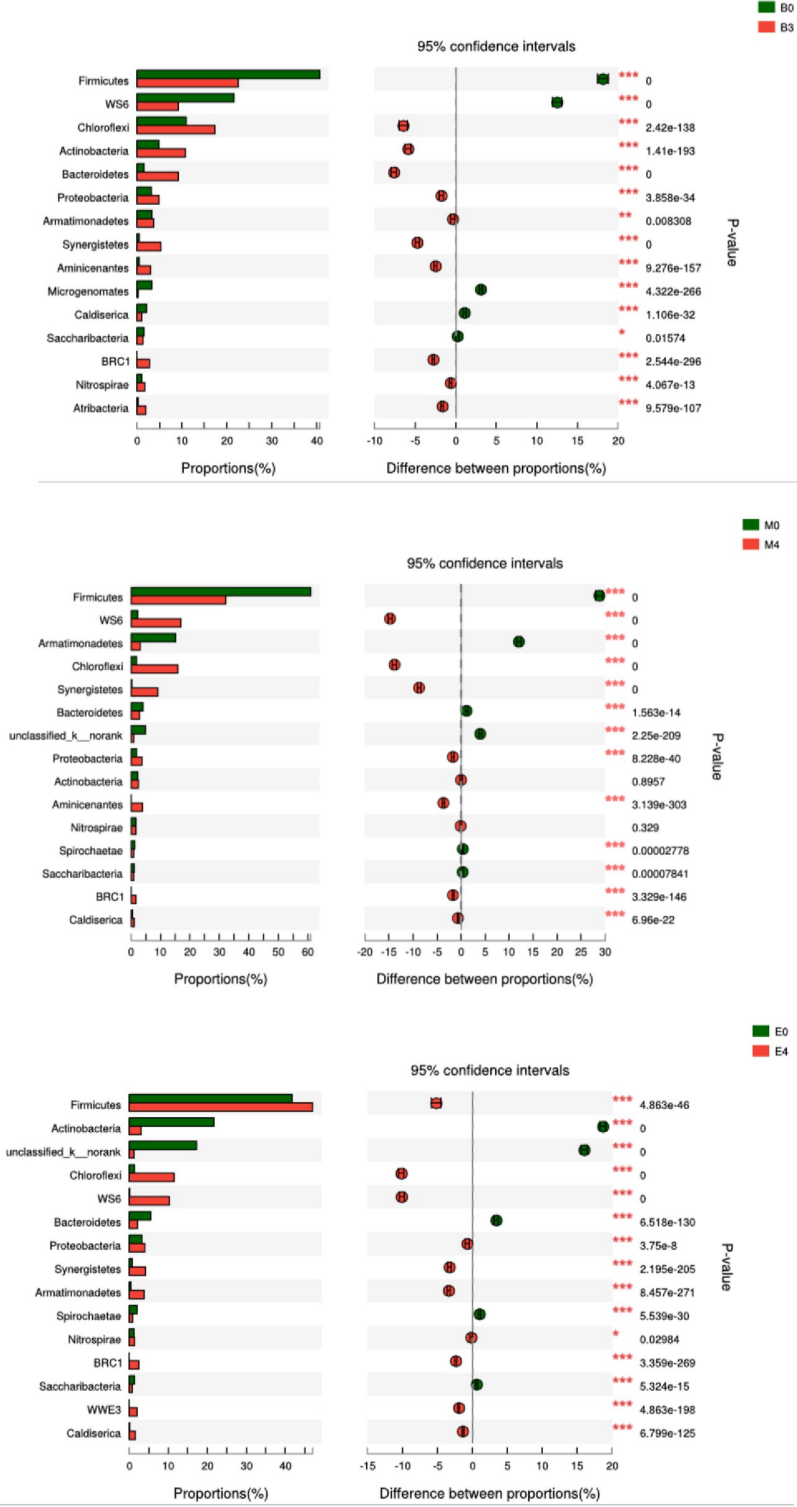
Fisher’s exact text bar plot for bacterial community on phyla level.

The co-substrates with different ratios had also changed the microbial composition. Microbial community analysis was performed to further investigate the composition of the microbial community. Fig 5 shows Fisher’s exact text bar plot for bacterial community on phyla level in digester B0, B3, M0, M4, E0 and E4. In general, there were six dominant phyla were identified in these digesters i.e. *Firmicutes*, *Chloroflexi*, *Actinobacteria*, *Bacteroidetes*, *Proteobacteria*, and *Synergistete*. *Firmicutes* is a very common bacterium existing in anaerobic sludge. The main function of *Firmicutes* is producing extracellular enzymes such as protease, lipase and cellulase, which were closely related to the degradation of organic compounds and the formation of VFA [38]. In B3, M4 and E4, with the bentonite added, the percentages of Firmicutes in digester B3 and M4 decrease by 18.28% and 28.83%, while the E4 increases by 5.03%. *Chloroflexi* was one of the numerically important carbohydrate- hydrolyzing bacterial groups in anaerobic sludge digester, which is a strict anaerobic multicellular filamentous microbe, and it can degrade glucose [39]. The abundance of *Chloroflexi* were significantly improved after adding bentonite into system, which can be used to explain the higher VS removal of bentonite-added groups. *Bacteroidetes* also played a very important role in the hydrolysis and acidification process, mainly involving the degradation of proteins, fats, cellulose and other polysaccharides [40]. *Actinobacteria* was found to be dominant hydrolytic population in AD of WAS [41]. It prefers to live in a slightly acidic environment for the fast of hydrolysis of organic compounds. Due to the more KW proportion addition, trial E presented the lower pH value among all trials, which means a fast-hydrolytic rate of this trial. The fast-hydrolytic rate and the lower pH value in this trial contributed accumulation of VFAs. Addition of bentonite into digester (E4) decreased the richness of *Actinobacteria* and thus reduced the hydrolytic rate of substrates, which further eased the problem of VFAs accumulation. *Proteobacteria* is typical bacteria existing in the AD reactor, and it is reported to be the most dominant bacteria flora in sludge of wastewater treatment [42]. It also plays an important metabolic function in AD process, for its utilization of glucose, propionate, butyrate and other small molecular compounds. *Synergistetes* belongs to acetogens, which are capable of acetogenesis and accelerate the transfer of VFAs to acetate e.g. CH_3_CH_2_COOH + 2H_2_O → CH_3_COOH + CO_2_ + 3H_2_ [43].

(2) Pyrosequencing analysis of archaea community

**Fig 6:**
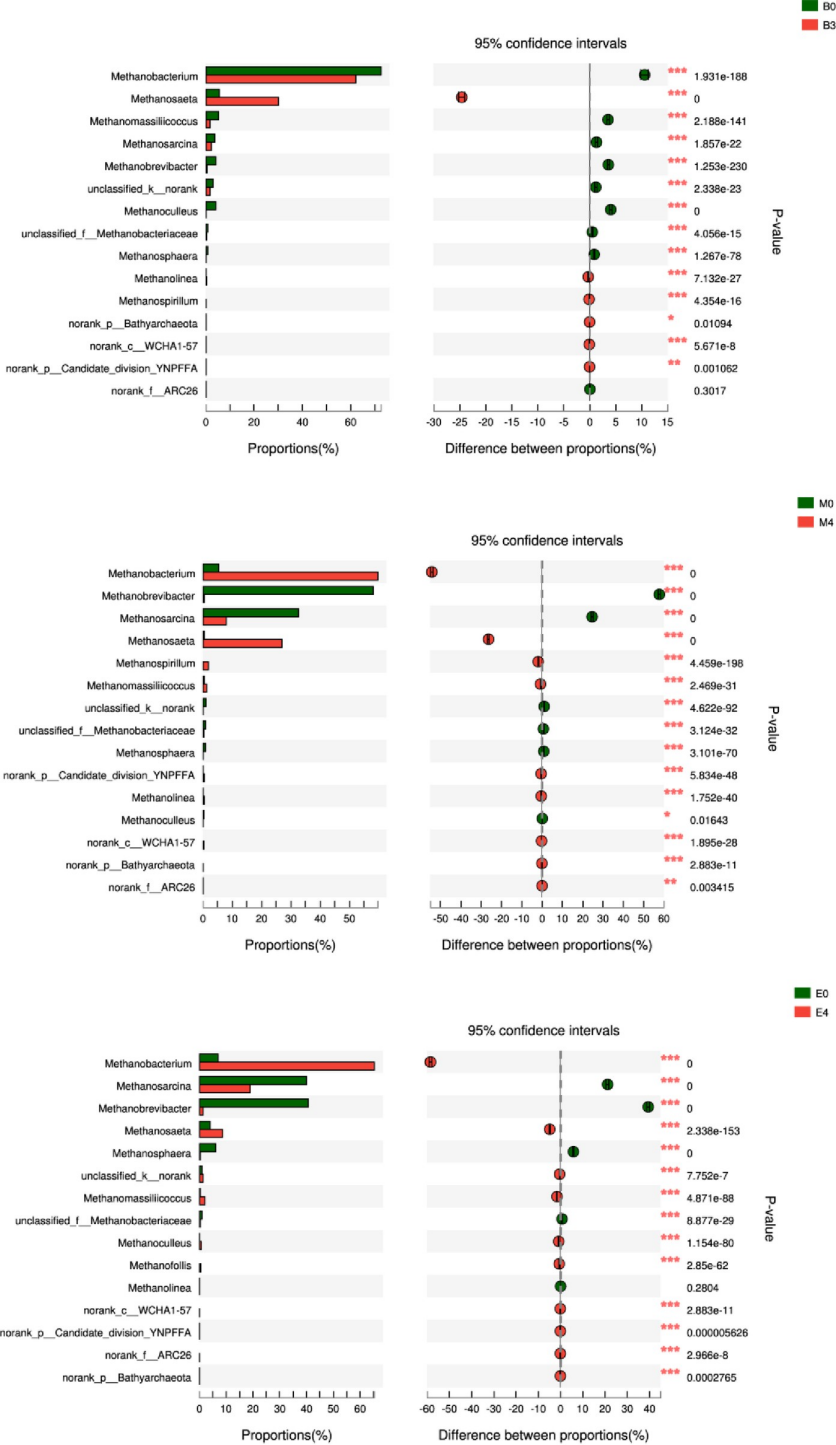
Fisher’s exact text bar plot for archaeal community on genus level.

Through biodiversity of archaeal communities’ analysis, B0, B3, M0, M4, E0 and E4 have an equal sequencing depth (33716 reads) after subsampling. To make further investigation, the archaeal taxonomy analysis was conducted on genus level, and the results are shown in Fig 6. From this figure, we can see that *Methanobacterium*, *Methanosarcina*, *Methanobrevibacter and Methanosaeta* were prevailed. *Methanobacterium* is a kind of hydrogenotrophic methanogenic archaeon, which can only use formate and glycol as substrates to product methane in extreme strict anaerobic environment [44]. Compared to B0, *Methanobacterium* in digester M0 and E0 significantly decreased much more significantly with the increase of the KW proportion in co-substrates, which was due to the huge amount of organic contents in these two digesters. *Methanobacterium* is very sensitive to pH and tended to present a decrease at low pH value. It can be seen from Fig 6 that addition of bentonite into system brought a tremendous growth of *Methanobacterium*, indicating that the VFAs inhibition problem could be eased by bentonite addition. *Methanosaeta* is a kind of methanogens which can use HAc in producing methane, and it plays the most important role in methanogenic process (accounts for about 70% of methane production) [45]. It can be seen from the figure that addition of bentonite into digester could improve the *Methanosaeta* richness, which can be applied to illustrate the higher methane yields produced by bentonite-added groups. *Methanosarcina* is the most diverse methanogens and can use acetate, H_2_/CO_2_ and methylated one-carbon compounds as substrate for methane production through three metabolic pathways. *Methanobrevibacter* is a kind of acidophilic methanogens, of which the main function is producing methane by degrading hemicellulose [46]. After adding bentonite into system, *Methanobrevibacter* tended to extinct, which was due to the buffer capacity of bentonite.

Through the analysis of microbial community, we can draw a conclusion that the addition of bentonite plays a critical role of moderating microbial community in AD process, which could reduce microbial richness by selecting some useless microbes and optimizing the structure of microbes.

## 4. Conclusion

Enhanced anaerobic co-digestion of WAS and KW by adding bentonite was investigated. Methane production and the digestion performance, as well as the enhancement mechanism were focused. The results showed that the addition of bentonite decreased the lag phase of the methane production, buffered acids accumulation, and promoted organics’ degradation. The highest specific methane production of 165.3 mL/g VS was achieved in M4 (WAS/KW = 1:2, based on VS, Bentonite dosage = 2.0 g/g VS). The more KW is applied in co-substrate mixture, the longer lag phase in gas production will be, but adding bentonite shortened the lag phase. Variation of pH and VFAs’ concentrations showed that all the digesters’ pH value can recover to 7.0–8.0 of the digestion, while the VFAs concentrations fell into appropriate ranges at the final stage, except E0. The adsorption capacities of bentonite to different volatile acids including acetic acid (HAc), propionic acid (HPr), and butyric acid (HBu) were examed and it presented good ability in acids adsorption, indicating that the adsorption effect brought by bentonite played an important role in improving of digestion performances. However, the reduced amounts of VFAs under bentonite addition were far more than the adsorption capacities of the added bentonite. Through analysis of bacteria and archaea community, it is confirmed that the bentonite addition was also helpful for maintaining microbial community stable and optimizing the structure of microbes.
